# Gabapentin to achieve HIV viral load suppression in people with risky drinking in Mbarara, Uganda: study protocol for a randomized, double-blinded, placebo-controlled trial (GRAIL)

**DOI:** 10.21203/rs.3.rs-6856284/v1

**Published:** 2025-10-15

**Authors:** Ve Truong, Allen Kekibiina, Winnie Muyindike, Natalia Gnatienko, Judith I Tsui, Nneka I Emenyonu, Debbie M Cheng, Judith Hahn, Olivia Allison, Matthew J Bullard, Karsten Lunze, Jeffrey H Samet

**Affiliations:** Boston Medical Center; Mbarara University of Science and Technology; Mbarara University of Science and Technology; Boston Medical Center; University of Washington; University of California San Francisco; Boston University School of Public Health; University of California San Francisco; Boston Medical Center; Boston University School of Public Health; Boston Medical Center; Boston Medical Center

**Keywords:** Uganda, HIV, Alcohol Use, Viral Load Suppression, Gabapentin, RCT

## Abstract

**Background:**

Ending the HIV epidemic requires achieving HIV viral load (HVL) suppression for key populations including those with risky drinking. Gabapentin can decrease alcohol use and thus holds potential for improving medication adherence and HIV viral load suppression for individuals with HIV and risky drinking.

**Methods:**

To describe the protocol for arandomized, double-blinded, placebo-controlled trial, (GRAIL [Gabapentin to Reduce Alcohol and Improve viral Load suppression]), to test the efficacy of gabapentin versus placebo on achieving undetectable HIV viral load among people with HIV with risky drinking. We recruit and randomize 300 people with detectable HIV viral loads (>= 200 copies/ml) and risky drinking. Participants are randomized 1:1 to receive either gabapentin (1800 mg/day target dose) or placebo for 3 months in a double blind design. Both arms receive a 5-minute evidence-based counseling session aimed at reducing alcohol use. The primary outcome is undetectable viral load (<200 copies/ml) at 3 months. Other secondary outcomes include undetectable viral load at 6 and 12 months, biomarker-confirmed recent alcohol consumption, antiretroviral therapy (ART) adherence, engagement in HIV care and pain severity. This study takes place in Mbarara, Uganda, as the country ranks among the top five for alcohol consumption globally and has 1.4 million people living with HIV.

**Discussion:**

GRAIL tests the efficacy of gabapentin, a medication to decrease alcohol use to achieve HIV viral load suppression. This trial may identify a treatment strategy to prevent the transmission of HIV and improve health outcomes among a high risk HIV population, specifically those with risky drinking.

**Trial registration:**

This trial has been registered at ClinicalTrials.gov (NCT05443555) on June 29, 2022. https://clinicaltrials.gov/study/NCT05443555?term=NCT05443555&rank=1

## Background

Over 1.4 million people in Uganda are living with HIV. Among this group, 90% are aware of their HIV status, 84% report receiving antiretroviral therapy (ART), and 79% of people with HIV (PWH) have achieved HIV viral load (HVL) suppression [1]. These statistics represent great progress, yet the country is facing barriers on its path to achieving the UNAIDS 95–95-95 HIV targets (i.e., detection, on medication, HVL suppression) [2]. The prevalence of heavy drinking is high in Uganda; its per capita alcohol consumption of 12 liters of alcohol per year places Uganda at the top of the list among African countries for alcohol consumption[3] and in the top five globally [4]. Alcohol consumption has negative impacts across the HIV care cascade, including effects on engagement in HIV care (i.e., individuals who are “Linked and Retained”), medication adherence, and HVL suppression [5, 6]. An intervention targeting alcohol consumption in PWH with heavy alcohol use and without HVL suppression has the potential to improve HIV outcomes such as medication adherence and HVL suppression. Increasing viral suppression rates could decrease the incidence of new HIV infections exemplifying “treatment as prevention”. Gabapentin has been shown to be an effective treatment for alcohol use disorder [7–10] and neuropathic pain [11], but its impact on outcomes in PWH with heavy alcohol use has not been evaluated [7–10]. Uganda’s high HIV and alcohol use burden make this setting suitable for studying gabapentin in this population.

This paper describes the protocol of the Gabapentin to Reduce Alcohol and Improve viral Load suppression (GRAIL) – Promoting “Treatment as Prevention”, randomized, double-blinded, placebo-controlled clinical trial among 300 people with unsuppressed HIV and risky drinking to evaluate the efficacy of gabapentin in promoting HVL suppression.

## Methods

GRAIL is a randomized, double-blinded, placebo-controlled clinical trial designed to evaluate the efficacy of gabapentin versus placebo in achieving undetectable HVL in 300 PWH who engage in recent risky drinking. Risky drinking is operationalized as a positive Alcohol Use Disorders Identification Test - Concise (AUDIT-C) and urine ethyl glucuronide (EtG) test. Participants are randomized 1:1 to receive either gabapentin or placebo for 3 months; both arms receive a brief intervention to reduce alcohol use.

### Study setting

**Figure 1:**
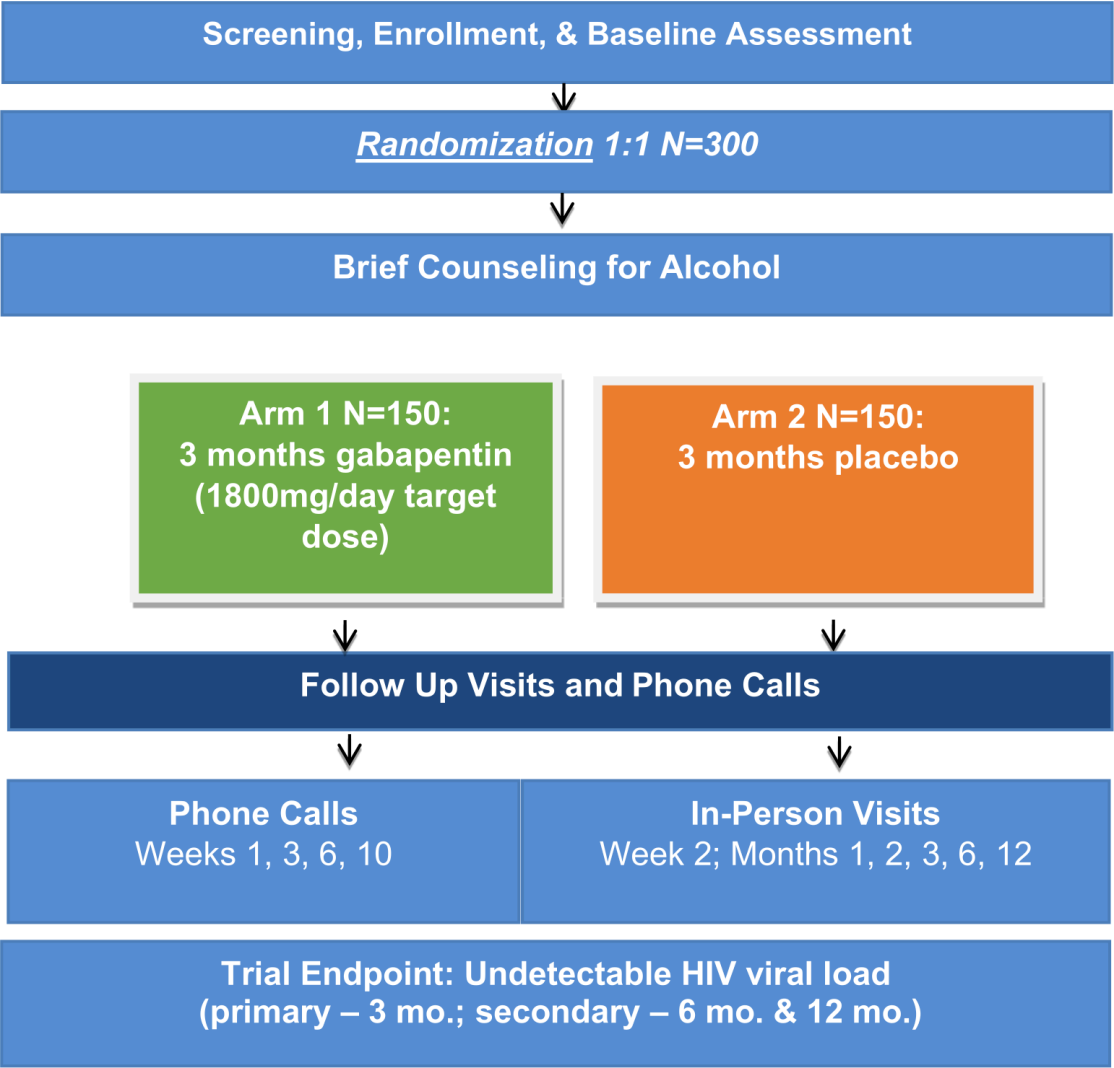
GRAIL study design

**Figure 2:**
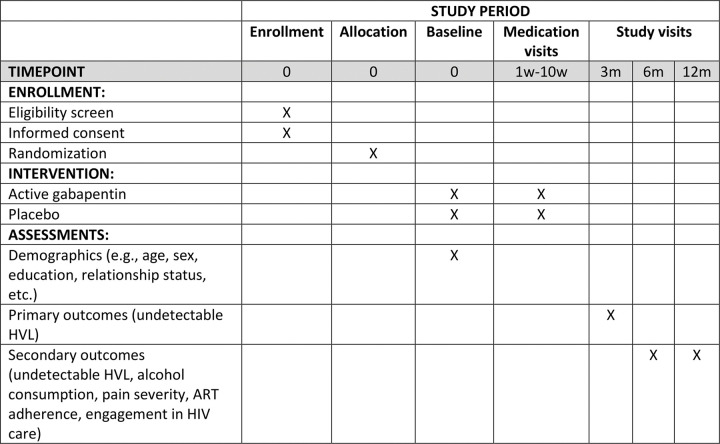
Schedule of enrollment, intervention, and assessments (SPIRIT figure)

The study is being implemented at Mbarara University of Science and Technology (MUST) in Mbarara, Uganda. MUST is a major educational, scientific, and clinical medical institution. Recruitment occurs at the Immune Suppression Syndrome (ISS) Clinic at the Mbarara Regional Referral Hospital in Uganda, HIV Care Clinics (e.g., Mbarara City Council Health Centre 4 and others within and outside the city and district), as well as through snowball recruitment. The ISS Clinic has provided HIV care since November 1998. The adult care section of the clinic currently cares for over 11,500 adults with HIV. The SPIRIT checklist of recommended items to address in a clinical trial protocol is available as Additional File 1. See Fig. 1 for study flow and Fig. 2 for the planned schedule of enrollment, intervention and assessments.

### Participants and recruitment

In Uganda, clinics typically initiate ART for patients newly diagnosed with HIV. Therefore, our criteria require that a participant has been diagnosed with HIV for at least six months, and their detectable HVL must be recorded at least six months post-diagnosis to strongly suggest non-adherence to ART. Participants are eligible if they meet the following additional criteria: 18 years or older; positive EtG urine test (300 ng/ml cutoff); able and willing to comply with all protocols and procedures; and living within 2 hours travel time of the study site. They are excluded for the following: not fluent in English or Runyankole; cognitive impairment resulting in inability to provide informed consent based on research assistant assessment; pregnant, planning to become pregnant in the next 3 months, or breastfeeding; taking gabapentin/pregabalin in past 30 days; taking any medication for alcohol use disorder; known hypersensitivity to gabapentin; enrolled in another HIV research study seeking viral load suppression; and has an unstable psychiatric illness. Study eligibility criteria are detailed in Table 1. A clinic staff member reviews electronic medical records to identify patients with upcoming clinic appointments who potentially meet eligibility criteria. Patients meeting the initial eligibility criteria (i.e., HIV diagnosis and most recent detectable HVL) are approached during their appointment to assess interest in study participation. Those interested are screened by a study research assistant (RA), who is a clinical officer, to determine further eligibility. In addition, the GRAIL study data manager and project coordinator consult with clinic counselors overseeing the non-suppression registry to identify patients with a recent detectable HVL test who were not captured through the initial screening process.

RAs either meets potential participants at the clinics following their routine clinic visit or makes an appointment to meet in person at a later date at one of the recruitment clinics. For participants unable to travel to the clinic, RAs provides transportation from their home to the clinic. RAs introduce the study to eligible participants, gauge their interest and explain the purpose of the study. If further eligibility is established, the RA seeks informed consent. Randomization follows the completion of the baseline assessment.

### Randomization

Randomization is conducted and monitored by the data management team. Participants are randomized in a 1:1 ratio using permuted blocks with a fixed block size of two, stratified by: i) self-reported alcohol use severity score (Alcohol Use Disorders Test (AUDIT) > 20 vs. ≤ 20) and ii) clinic site. We assumed participants with severe alcohol use may be less likely to reach HVL suppression over the study period than those who are drinking less alcohol.

Randomized group allocations are concealed using scratch-off cards. Before recruitment, the study pharmacist received a randomization list containing sequential card numbers for ordering and a unique 5-digit code (randomization result) indicating the medication assigned arm (gabapentin and placebo). Each scratch-off card displays only the card number, while the randomization code remains hidden until the participant scratches off the covering. The AUDIT score stratum is distinguished by the color of the scratch card (i.e., yellow for AUDIT > 20 and green for AUDIT ≤ 20).

During randomization, RAs present participants with the top four sequential scratch-off cards from the appropriate AUDIT pile. Participants select one card, scratch it off, and reveal the randomization result. Based on the result, the RA provides the corresponding medication package labeled with that unique randomization number. The next sequential card is then added to the pile, ensuring that four cards are consistently available for subsequent participants.

### Intervention

Eligible participants are then randomly assigned into one of two study arms: 1) gabapentin (up to 600mg [2 pills] three times a day, i.e., 1800mg/day target dose) for 3 months vs. 2) placebo for 3 months. At baseline, all participants will receive brief (5 minutes) evidence-based counseling based on the severity of their alcohol use disorder (per DSM-5) from the study assessment. This counseling approach was adapted from the NIAAA Clinician’s Guide [12]. Participants who continue to meet the criteria for alcohol use disorder at 3 months are offered additional alcohol counseling sessions with GRAIL study counselors. At study initiation, the medication is provided by trained clinical officers, who instruct participants in proper medication administration and adherence.

Study medications were purchased from the Gama Pharmaceuticals (U) Ltd and EVA Pharma. Placebo medications do not contain active ingredients. The placebo and active study medications are indistinguishable by packaging, appearance and taste to allow for double blinding. The study pharmacist packages study medications into paper envelopes. Participants and study team members are blinded to assigned treatment.

Medication dosing is based on Mason et al [8]. Dosing is titrated up over 3 weeks, starting with a daily dose of 300mg (1 capsule/day) in week 1, followed by a daily dose of 900mg (3 capsules/day) in week 2, up to a target daily dose of 1800mg (6 capsules/day) in week 3. The target dose of 1800mg per day is sustained from weeks 3 through day 4 of week 12. Then, the dose is tapered down to 900mg in days 5–7 of week 12, and medication is discontinued at the end of week 12. If a participant in any group experiences an adverse event, the symptom is documented and monitored at each study visit. If reductions in medication are required due to side effects, the participant will be advised to reduce their dose to that of the prior week. The GRAIL medication schedule can be seen in Table 2. Participants are instructed to bring any unused capsules to their upcoming in-person study visit.

### Adherence

Medication adherence is assessed at each medication check-in using capsule counts and self-report. At each medication check-in visit, medication instructions are reviewed with the participant and strategies to support adherence are discussed. To further increase medication adherence, a text message is sent out daily reminding participants to take their study medication. To ensure participant privacy and to accommodate those who may not wish to disclose their involvement in a research study or share their phones, the message reads: “Please remember GRAIL.” Participants are able to reduce the frequency to 2 days per week or opt out of text message reminders entirely at any time throughout the study.

### Study outcomes

The primary outcome is undetectable HVL (< 200 copies/ml) at 3 months, with secondary outcomes being undetectable HVL at 6 and 12 months. In order to explore potential mechanisms by which gabapentin may lead to HVL suppression, we assess the impact of gabapentin compared to placebo on the following secondary outcomes: a) alcohol consumption; b) pain severity; c) ART adherence; and d) engagement in HIV care.

### Questionnaires

Participants are assessed at baseline, 3, 6, and 12 months post enrollment, along with shorter medication visits by phone at weeks 1, 3, 6 and 10 and shorter medication visits in-person at weeks 2, 4 and 8. All follow-up study visits take place at the clinic from where the participant was recruited or by phone.

Participants receive a meal at each study visit, along with either 1 kg of rice and one bar of soap, or two bars of soap, as compensation. Transport costs are also reimbursed: 20,000 UGX (5.33 USD) for those living within 20 km, 30,000 UGX (8 USD) for 20–40km, and up to 45,000 UGX (12 USD) for 40–60km from the study site.

The study has employed a driver to provide transportation to the clinic for participants who require a ride to their study visit appointments.

### Questionnaires

Components of the baseline and follow-up assessments are listed in Table 3. At each in-person study visit, RAs measure and record participants’ weight and blood pressure. Height is measured at the baseline visit. Over the course of the study, we review medical charts to obtain HIV treatment history. Participants are asked to provide consent so that their data may be used for potential future ancillary studies.

### Laboratory testing and results

Blood is collected throughout the study. CD4 count and creatinine are tested at baseline. Phosphatidylethanol (PEth, an alcohol biomarker) [13], is tested at baseline and 3 months. HIV viral loads are tested at 3, 6, and 12 months. Participants with a creatinine clearance of < 60mL/min have their study medication dose reduced per clinical recommendations for prescribing gabapentin for patients with chronic kidney disease. Additionally, at the 3-month study visit, all participants are asked to provide blood for an HIV drug resistance test, however the test is only conducted for those with a detectable HVL at 3 months to assess if the HIV is resistant to that participant’s ART regimen.

Urine samples are collected during screening to test for pregnancy and alcohol use, the latter by using an ethyl glucuronide (EtG) dip test. An EtG dip test is a type of urine test used to detect the presence of alcohol. In this study, we use a test with an 80-hour detection window and a 300 ng/mL cutoff. Additionally, urine is collected for women during in-person medication check-ins and at 3 months to confirm the participant is not pregnant. Participants found to be pregnant will discontinue their study medication but will continue follow-up assessments for the duration of the study.

### Retention of study participants

Participants are asked to provide contact information for 2–3 alternative contacts (e.g., friends and family) who can assist in the event of being lost to follow-up. Efforts to improve retention for all participants include the provision of refreshments during study visits, reimbursements for travel expenses, and conducting home visits when necessary.

### Adverse events and safety monitoring

At baseline, participant symptoms are assessed to document any chronic conditions or symptoms that existed prior to the introduction of study medication. These are documented on the baseline event form and are reviewed and compared to reported adverse events throughout the study. During follow-up, symptoms are assessed weekly for the first month, and biweekly thereafter while the participants are administered study medication. If a participant experiences a severe or medically significant but not immediately life-threatening symptom, the RA increases monitoring frequently for safety, and the team considers adjusting the medication dose or stopping it. Participants may be unblinded if there is an urgent medical need, as determined by the clinician evaluating the participant. If a participant is unblinded, study medication may be discontinued if deemed medically necessary.

Any event that meet the criteria for an adverse event (AE), serious adverse event (SAE), or unanticipated problem is recorded. All events are reviewed by a study physician. AEs and SAEs are periodically presented to and reviewed by an external Data and Safety Monitoring Board (DSMB), the IRBs, and the Uganda’s National Drug Authority.

### Data management

The Biostatistics and Epidemiology Data Analytics Center at Boston University provides statistical collaboration in the design and analysis of the GRAIL trial. Electronic questionnaires include programmed skip patterns and range checks, minimizing errors at data capture. Participant screening, assessment, tracking, and randomization utilize Research Electronic Data Capture (REDCap) mobile.

### Analytic methods

The study uses an intention-to-treat analysis that includes all participants according to their randomized assignment. Descriptive statistics are calculated for variables at baseline and each follow-up time to assess whether there appear to be any differences across treatment arms. Spearman correlation analyses are performed to identify pairs of variables that may be collinear. In addition, the variance inflation factor is assessed to detect possible collinearity.

The primary outcome for this study is undetectable HVL (< 200 copies/ml) at 3 months. The primary analysis uses multivariable logistic regression analyses to control for the randomization stratification factors to improve efficiency (i.e., AUDIT > 20, clinic site). If any baseline factors appear to differ by randomized group, additional sensitivity analyses will be conducted controlling for these factors to assess for potential confounding. Other binary outcomes (e.g., heavy alcohol consumption [defined as PEth ≥ 50 ng/mL], adherence to ART [defined as taking ≥ 80% of prescribed medication via Visual Analog Scale], engagement in HIV care [defined as ≥ 1 HIV visit in the past 3 months]) will be analyzed using the same approach. Number of heavy drinking days (assessed using the Timeline Followback [TLFB] method) in the past month will be analyzed using negative binominal regression, to account for overdispersion in the data. Percentage of ART pills taken (assessed by self-report), a continuous outcome, will be analyzed using multiple linear regression if the data are normally distributed. However, if the distribution is skewed, transformations of the data will be performed (e.g., log transformation). If an appropriate transformation is not identified, a median regression model will be used. A secondary analysis will be conducted using a per protocol approach that includes only those participants who were adherent to their assigned intervention (i.e., defined as taking at least 80% of the assigned medication), as determined by self-report (i.e., Visual Analog Scale).

Given the repeated measures of our primary outcome (i.e., 3, 6, and 12 months), additional exploratory analyses using longitudinal regression models will be used to incorporate repeated measures for each outcome in the same model and will test for possible gabapentin by time interactions. For the dichotomous undetectable HIV viral load outcome, we will use generalized estimating equations (GEE), with an autoregressive working correlation matrix, a logit link; standard errors will be based on the empirical-sandwich estimator. Longitudinal analyses of heavy alcohol consumption will be analyzed using the same approach. For number of heavy drinking days, we will use GEE negative binomial regression models. For continuous outcomes, such as percentage of ART taken, we will use generalized linear mixed effects models that include subject-specific random intercepts and slopes to account for the correlation due to having repeated observations from each subject.

### Sample size, power, and effect size

We present power calculations for the primary outcome HVL suppression at 3 months post randomization. The calculations assume an alpha level of 0.05 and that 300 participants are enrolled into the trial. The GRAIL trial was initially designed to be conducted in Russia, with our sample size justification derived from prior research conducted among the Russian population. Based on data from our previous study of Russian people with HIV and risky drinking [14], we expect 40% of control participants will have undetectable HVL at 3 months. Given this and assuming 15% loss to follow-up at 3 months (i.e., 255 evaluable subjects) the study will have 80% power to detect an absolute difference of 19% in the proportions with undetectable HVL at 3 months (i.e., 59% vs. 40% in the gabapentin and control arms, respectively) using a chi-square test with continuity correction. We anticipate larger effects may be observed in the GRAIL trial, which would result in even higher power. In the Russian study, among participants on ART who reduced alcohol consumption, approximately 76% had HVL suppression at the 3-month follow-up. If a similar proportion achieves HVL suppression in the gabapentin arm, the study would have > 99% power to detect this 36% difference between randomized groups (i.e., 76% vs. 40% in the gabapentin and control arms, respectively). The trial is adequately powered to detect differences between arms as small as 19%, which are still considered clinically meaningful.

### Missing data

Participants who meet eligibility criteria and agree to participate will be compared with participants who were determined to be eligible but declined enrollment on data captured during eligibility assessment. Missing data patterns will be evaluated including the frequency and percentage of participants missing for each variable and the distribution of the number of variables missing for participants. In addition, data collected to the point of loss to follow-up will be compared to the data of those who complete the study to examine missing data mechanisms. The proposed study has accounted for a 15% random non-informative loss to follow-up and will still have sufficient power with this potential loss in size. It may be reasonable to assume that data are missing at random, albeit not completely. If this is the case, multiple model-based imputation methods will be applied to account for the missing data, if needed. We will also consider the use of pattern mixture models, which is applicable when the data are not missing at random. Pattern mixture models involve classifying subjects based on their longitudinal missing data patterns and assessing whether there appears to be an interaction between a randomized group and missing data pattern.

### Protection of study data

All study data are captured electronically on computers via a secure, web-based data capture system. The study data are located on a secure server within the Boston University Medical Center domain. Identifiers needed to track participants are kept separate from research data.

### Data safety monitoring board (DSMB)

To ensure the safety of the participants and the validity and integrity of the data, an external DSMB was established to assume oversight of the study. The DSMB operates independently of the study sponsor and study investigators and its members have no competing interests that could affect their objectivity in monitoring the safety and integrity of the study. The Board meets every 6 months and is charged with evaluating the quality of trial administration, monitoring safety issues, and providing guidance on scientific, methodological, and ethical issues. Specifically, the Board reviews investigators’ plans and processes for identifying individual or patterns of adverse events and reviews accumulating safety data. They may use results from an interim analysis to recommend that the study proceed as planned, be temporarily suspended, or be terminated. The DSMB charter is available as Additional File 2.

### Auditing

The research team has employed a Study Monitor to conduct monitoring visits at study recruitment sites and research office. These typically occur annually in order to review study documents, and collaborate with the research team to ensure compliance with protocols and regulations. The study monitor provides input without influence from investigators or sponsor.

### Participant graduation, withdrawal, and disenrollment

Participant disenrollment can occur at any time during the study upon a participant’s request to withdraw from study participation. Graduation for participants occurs after all procedures for the 12-month follow-up visit are complete or when a participant is withdrawn or terminated from the study. At their final study visit, participants receive a graduation certificate in appreciation of their contribution to the research.

### Dissemination plans

De-identified trial data are uploaded into the NIAAA Data Archive biannually. Study results will be submitted for publication in peer-reviewed journals relevant to the research topic. Abstracts will be submitted to national and international conferences relevant to the field.

## Discussion

The GRAIL study seeks to establish whether gabapentin can achieve HVL suppression among PWH with risky drinking. The study is being conducted in a setting of high HIV prevalence with unmet needs for treatment and prevention of alcohol use. If shown to be effective, this highly generalizable pragmatic approach, with the potential of rapid deployment of gabapentin due to its affordability and widespread availability, can be added to the HIV prevention toolkit and implemented within existing medical infrastructure.

### Trial status

The study was initially approved by the BUMC IRB on August 13, 2022 (version 1.1). The current version was approved on July 22, 2024 (version 1.6). Recruitment started on November 20, 2023 and is expected to complete in May 2026.

## Supplementary Material

Supplementary Files

This is a list of supplementary files associated with this preprint. Click to download.

• Table3.docx

• AdditionalFile1.SPIRITChecklist.docx

• GRAILDSMBCharterv1.1Feb2023.docx

Table 3 is available in the Supplementary Files section.

## Figures and Tables

**Table 1:** GRAIL eligibility criteria

**Inclusion Criteria**
18 years or older
Having an HIV diagnosis for at least 6 months (assessed via medical charts)
Current (within 2 months) detectable HIV viral load at least 6 months after HIV diagnosis (assessed via medical charts or study test)
Positive Ethyl Glucuronide (EtG) urine test (300 ng/ml cutoff)
Able and willing to comply with all study protocols and procedures
Living within 2 hours travel time of the study site
**Exclusion Criteria**
Not fluent in English or Runyankole
Cognitive impairment resulting in inability to provide informed consent based on research assistant assessment
Pregnancy, planning to become pregnant in next 3 months, or breast feeding (pregnancy assessed via study test)
Taking gabapentin/pregabalin in past 30 days (self-report)
Taking any medication for alcohol use disorder (self-report)
Known hypersensitivity to gabapentin (self-report)
Enrolled in another HIV research study seeking viral load suppression (self-report)
Unstable psychiatric illness (i.e., answered yes to any of the following: past three month active hallucinations; mental health symptoms prompting a visit to the ED or hospital; mental health medication changes due to worsening symptoms; presence of suicidal ideations)

**Table 2:** GRAIL medication dosing schedule

Week 1: 1 capsule per day; 7 capsules total							
	Day 1	Day 2	Day 3	Day 4	Day 5	Day 6	Day 7
Morning	1 capsule	1 capsule	1 capsule	1 capsule	1 capsule	1 capsule	1 capsule
Afternoon							
Evening							
Total	1	1	1	1	1	1	1
Week 2: 3 capsule per day; 21 capsules total							
	Day 1	Day 2	Day 3	Day 4	Day 5	Day 6	Day 7
Morning	1 capsule	1 capsule	1 capsule	1 capsule	1 capsule	1 capsule	1 capsule
Afternoon	1 capsule	1 capsule	1 capsule	1 capsule	1 capsule	1 capsule	1 capsule
Evening	1 capsule	1 capsule	1 capsule	1 capsule	1 capsule	1 capsule	1 capsule
Total	3	3	3	3	3	3	3
Week 3, 4, 5, 6, 7, 8, 9, 10, 11: 6 capsule per day; 42 capsules total							
	Day 1	Day 2	Day 3	Day 4	Day 5	Day 6	Day 7
Morning	2 capsules	2 capsules	2 capsules	2 capsules	2 capsules	2 capsules	2 capsules
Afternoon	2 capsules	2 capsules	2 capsules	2 capsules	2 capsules	2 capsules	2 capsules
Evening	2 capsules	2 capsules	2 capsules	2 capsules	2 capsules	2 capsules	2 capsules
Total	6	6	6	6	6	6	6
Week 12: 33 capsules total							
	Day 1	Day 2	Day 3	Day 4	Day 5	Day 6	Day 7
Morning	2 capsules	2 capsules	2 capsules	2 capsules	1 capsule	1 capsule	1 capsule
Afternoon	2 capsules	2 capsules	2 capsules	2 capsules	1 capsule	1 capsule	1 capsule
Evening	2 capsules	2 capsules	2 capsules	2 capsules	1 capsule	1 capsule	1 capsule
Total	6	6	6	6	3	3	3

## Data Availability

Data and materials will be provided upon reasonable requests to the corresponding author.

## Abbreviations

AE: Adverse event
ART: Antiretroviral therapy
AUDIT: Alcohol Use Disorders Identification Test
AUDIT-C: Alcohol Use Disorders Identification Test- Concise
BPI-SF: Brief Pain Inventory- Short Form
CD4: Cluster of differentiation 4
CESD-10: Center of Epidemiologic Studies Depression Scale, 10-item version
DSMB: Data Safety Monitoring Board
DSM-5: Diagnostic and Statistical Manual of Mental Disorders
EtG: Urine ethyl glucuronide test
GAD-7: Generalized Anxiety Disorder-7
GEE: Generalized estimating equations
HVL: HIV viral load
IRB: Institutional Review Board
ISS: Immune Suppression Syndrome Clinic
MUST: Mbarara University of Science and Technology
NIAAA: National Institute on Alcohol Abuse and Alcoholism
PEth: Phosphatidylethanol
PWH: People with HIV
RA: Research assistant
REDCap: Research Electronic Data Capture
SAE: Serious adverse event
TLFB: Timeline Followback
UGX: Ugandan Shilling
USD: United States dollar
